# Development and validation of nomograms for predicting survival probability of patients with advanced adenocarcinoma in different EGFR mutation status

**DOI:** 10.1371/journal.pone.0220730

**Published:** 2019-08-16

**Authors:** Hsi-Chieh Chen, Elise Chia-Hui Tan, Chih-Hsien Liao, Zhong-Zhe Lin, Ming-Chin Yang

**Affiliations:** 1 Institute of Health Policy and Management, College of Public Health, National Taiwan University, Taipei, Taiwan; 2 National Research Institute of Chinese Medicine, Ministry of Health and Welfare, Taipei, Taiwan; 3 Institute of Hospital and Health Care Administration, National Yang-Ming University, Taipei, Taiwan; 4 School of Health Care Administration, Taipei Medical University, Taipei, Taiwan; 5 Departments of Oncology, National Taiwan University Cancer Center, National Taiwan University, Taipei, Taiwan; 6 Department of Internal Medicine, College of Medicine, National Taiwan University, Taipei, Taiwan; National Cancer Centre Singapore, SINGAPORE

## Abstract

**Introduction:**

Molecular markers are important variables in the selection of treatment for cancer patients and highly associated with their survival. Therefore, a nomogram that can predict survival probability by incorporating epidermal growth factor receptor mutation status and treatments for patients with advanced adenocarcinoma would be highly valuable. The aim of the study is to develop and validate a novel nomogram, incorporating epidermal growth factor receptor mutation status and treatments, for predicting 1-year and 2-year survival probability of patients with advanced adenocarcinoma.

**Material and methods:**

Data on 13,043 patients between June 1, 2011, and December 31, 2014 were collected. Seventy percent of them were randomly assigned to the training cohort for nomogram development, and the remaining 30% assigned to the validation cohort. The most important factors for constructing the nomogram were identified using multivariable Cox regression analysis. The discriminative ability and calibration of the nomograms were tested using C-statistics, calibration plots, and Kaplan-Meier curves.

**Results:**

In the training cohort, 1-year and 2-year OS were 52.8% and 28.5% in EGFR(-) patients, and 73.9% and 44.1% in EGFR(+) patients, respectively. In EGFR(+) group, factors selected were age, gender, congestive heart failure, renal disease, number of lymph node examined, tumor stage, surgical intervention, radiotherapy, first-line chemotherapy, ECOG performance status, malignant pleural effusion, and smoking. In EGFR(-) group, factors selected were age, gender, myocardial infarction, cerebrovascular disease, chronic pulmonary disease, number of lymph node examined, tumor stage, surgical intervention, radiotherapy, ECOG performance status, malignant pleural effusion, and a history of smoking. Two nomograms show good accuracy in predicting OS, with a concordance index of 0.83 in EGFR(+) and of 0.88 in EGFR(-).

**Conclusions:**

The survival prediction models can be used to make individualized predictions with different EGFR mutation status and a useful tool for selecting regimens for treating advanced adenocarcinoma.

## Introduction

Lung cancer has the highest incidence and is the leading cause of death among all carcinomas worldwide, with non-small cell lung cancer (NSCLC) accounts for approximately 83% of all cases of lung cancer[1], and adenocarcinoma is the most common type of histology (approximately 40%).[2, 3] Unfortunately, two-thirds of the patients were already in advanced stages (stage IIIB/IV) at the time of diagnosis, [4] therefore, prognosis assessment of adenocarcinoma patients is the first step toward making informed medical care decisions. This will help orient physicians with regard to the selection of treatment options for individual patients.[5] However, most existing models were derived from clinical trials that included only a small number of patients with homogeneous patient characteristics, thereby limiting the generalizability of their findings to real-world settings.[6–8] Other models include variables that are not readily available in routine clinical practice or rely on tests that can vary considerably over time (e.g. tests for glucose, albumin).[9]

Some researchers have incorporated in their prognostic models with treatments such as chemotherapy [10–12], chemotherapy in combination with vascular endothelial growth factor (VEGF)-tyrosine kinase inhibitor (TKI) [7], and epidermal growth factor receptor (EGFR)-TKI.[13–15] Nonetheless, those models are limited by a lack of information pertaining to the patients’ EGFR molecular profile, which has been firmly proved to have a significant impact on the overall survival (OS) and progression-free survival (PFS) of NSCLC patients.[16–18]

Thus, this study aimed to develop and validate a set of prognostic models incorporating demographic, clinical, and treatment-related characteristics according to the EGFR mutation status of Asian patients with advanced stage adenocarcinoma.

## Material and methods

### Data source and patient population

The study population comprised all individuals with incidental adenocarcinoma registered in the nationwide population-based Taiwan Cancer Registry (TCR) data during the period of 2011–2014. The TCR was established by the Ministry of Health and Welfare in Taiwan since 1979 and contained patients with newly diagnosed malignant tumors in hospitals with 50 or more beds [19]. The other data used in this study was obtained from National Health Insurance (NHI) claims data released by the Health and Welfare Data Science Center, Ministry of Health and Welfare. This is a longitudinal database containing all the medical claims records of the insured individuals, including ambulatory visits, hospital admissions, procedures, and medications. To identify the demographic characteristics and survival status of each patient in the study, we linked TCR data, NHI Registry data and Causes of Death data using scrambled identification number of individuals.

We identified adult patients (≧18 years) with unique lung cancer tumor (International Classification of Disease for Oncology, 3rd edition (ICD-O-3) codes: C34.0-C34.9) with morphology codes indicating adenocarcinoma (ICD-O-3-M-8050, 8140, 8230, 8250–8255, 8260, 8310, 8323, 8333, 8480, 8481, 8490, 8550) registered in the Taiwan Cancer Registry (TCR) data file between June 1, 2011 and December 31, 2014. Among the patients with adenocarcinoma and those with an unknown date of cancer diagnosis were excluded. We also excluded those patients who had the death date before the diagnosed date (the date of pathological report provided). Then, all the patients were followed-up until the date of death or at the end of the study on December 31, 2016. The staging system used in this study was the 7^th^ edition of the TNM classification.[20] We then linked the Taiwan Cancer Registry data, NHI claims data, NHI Registry Data, and Cause of Death data for the years 2011–2016 to derive the individual and clinical characteristics of patients, treatment patterns, and survival status. The EGFR mutation status of all enrolled patients were also collected from the cancer registry data as to whether they were EGFR mutation positive [EGFR(+)] or wild-type [EGFR(-)].

The initial date of diagnosis was used as the index date of cohort entry. The generalizability of the model was assessed by allocating patients randomly into two cohorts: the training cohort and validating cohort. Seventy percent of the study patients were randomly assign to the training cohort while the remaining 30% of patients as the external validation cohort.

### Statistical analysis

#### Construction of nomogram

The 1-year and 2-year risk of death for patients with advanced stage adenocarcinoma were estimated using the Cox proportional hazard model. Survival time was calculated as the difference between the date of diagnosis and date of death or at the end of the study. Tumor characteristics, smoking and drinking history, and body mass index (BMI) were obtained from the Taiwan Cancer Registry Data. Demographic variables, included age, gender, comorbidities, and treatments received were obtained from the NHI Registry and claims data. Comorbidities were considered present if a diagnosis was recorded prior to the diagnosis of adenocarcinoma. Treatments included first-line therapies, radiotherapy, and surgery. Missing values for any variable were coded as a separate missing/unknown category.

To construct a set of novel nomograms, we aim to identify the most important factors, among all possible variables, that affect survival time of advanced adenocarcinoma patients. Therefore, we first performed univariate Cox regression to assess the association between each variable and death. Variables with statistical significance (p < .05) and clinical relevance were considered candidates for multivariable Cox proportional hazard regression model with stepwise process. A final model was selected according the Akaike information criterion (AIC) across different models, the lower AIC of the model suggesting better fit of the model.[21] The generalized R^2^ was used to compare the association between independent predictor and outcomes.[22, 23] The residual analysis was applied to examine the goodness-of-fit of model. The ratios of calculated Beta coefficients (BETAs) were used to determine the proportional prognostic effects of these variables in the nomogram and to transfer into score from 0–100. Statistical analyses were conducted using SAS version 9.4 for Windows (SAS, Cary, NC).

#### Validation and calibration of nomogram

A 2-step validation of nomogram was employed. First, the performance of the model used for predicting outcomes was evaluated by calculating the Harrell concordance index (C-index).[24] The value of the C-index ranged from 0.5 to 1.0, with 0.5 indicating random chance and 1.0 indicating perfect discrimination of model outcomes. Secondly, calibration plots of the nomogram for 1- and 2-year overall survival was drawn to compare predicted survival probability with the observed survival at 1 and 2-year intervals after diagnosis, after dividing patients into 10 groups, using quartiles of the predicted risk the cutoff points.

### Ethics standard

This study was approved by the Research Ethics Committee of National Taiwan University Hospital (approval number: 201605030W). All participants’ identifying information was scrambled in the study.

## Results

### EGFR mutations in lung adenocarcinoma among Asian patients

From the initial 13,818 patients with advanced adenocarcinoma, we excluded those who were EGFR(-) but received EGFR-TKI as first-line therapy (n = 771), and those who were ECOG equal to 5 points (n = 4). This left a total of 13,043 patients for analysis. The median follow-up time were 17.83 months (range: 0.1–68.0 months) for the full study cohort, 17.75 months (range: 0.1–68.0 months) for the training set, and 18.0 months (range: 0.47–67.8) for the validation set. Among these patients, 7,426 (56.9%) were EGFR(+) and 5,617 (43.1%) were EGFR(-).

Table 1 lists the demographic, clinical, pathological, and surgical characteristics of the training and validation cohorts. In the EGFR (+) group, 60.1% of patients were female, 94.1% of them had tumor stage IV and 16.8% of them had smoking history. In contrast, 38.1% of patients in EGFR (-) group were female and 24.8% of them had smoking history. The 1-year survival and 2-year survival status were significantly different between EGFR(+) and EGFR(-) (**S1 Fig**).

**Table 1:** Demographics and clinic pathologic characteristics of patients with advanced stage adenocarcinoma.

	Overall Cohort (n = 13,043)				Training Set (n = 9,130)				Validation set (n = 3,913)			
	EGFR(-)		EGFR(+)		EGFR(-)		EGFR(+)		EGFR(-)		EGFR(+)	
	N	(%)	N	(%)	N	(%)	N	(%)	N	(%)	N	(%)
N	5,617		7,426		3,950		5,180		1,667		2,246	
Age, mean (SD), y	64.0	(12.8)	66.0	(12.5)	64.0	(12.7)	66.0	(12.4)	65.0	(12.9)	66.0	(12.7)
≤60 y	2,181	(38.8)	2,481	(33.4)	1,549	(39.2)	1,714	(33.1)	632	(37.9)	767	(34.1)
61–70	1,446	(25.7)	1,933	(26.0)	1,060	(26.8)	1,374	(26.5)	386	(23.2)	559	(24.9)
71–80	1,383	(24.6)	1,958	(26.4)	933	(23.6)	1,363	(26.3)	450	(27.0)	595	(26.5)
>80 y	607	(10.8)	1,054	(14.2)	408	(10.3)	729	(14.1)	199	(11.9)	325	(14.5)
Gender												
Female	2,142	(38.1)	4,460	(60.1)	1,560	(39.5)	3,106	(60.0)	582	(34.9)	1,354	(60.3)
Male	3,475	(61.9)	2,966	(39.9)	2,390	(60.5)	2,074	(40.0)	1,085	(65.1)	892	(39.7)
Comorbidity (yes)												
MI	64	(1.1)	65	(0.9)	45	(1.1)	43	(0.8)	19	(1.1)	22	(1.0)
CHF	310	(5.5)	430	(5.8)	206	(5.2)	304	(5.9)	104	(6.2)	126	(5.6)
PVD	68	(1.2)	111	(1.5)	46	(1.2)	79	(1.5)	22	(1.3)	32	(1.4)
Cerebrovascular disease	592	(10.5)	854	(11.5)	400	(10.1)	599	(11.6)	192	(11.5)	255	(11.4)
Chronic pulmonary disease	2,125	(37.8)	2,454	(33)	1,518	(38.4)	1,726	(33.3)	607	(36.4)	728	(32.4)
Rheumatologic disease	74	(1.3)	88	(1.2)	53	(1.3)	60	(1.2)	21	(1.3)	28	(1.2)
Ulcer disease	1,051	(18.7)	1,409	(19)	735	(18.6)	981	(18.9)	316	(19)	428	(19.1)
Mild liver disease	408	(7.3)	543	(7.3)	275	(7.0)	380	(7.3)	133	(8.0)	163	(7.3)
DM	1,079	(19.2)	1,432	(19.3)	756	(19.1)	990	(19.1)	323	(19.4)	442	(19.7)
DM with chronic complications ^b^	294	(5.2)	387	(5.2)	207	(5.2)	282	(5.4)	87	(5.2)	105	(4.7)
Hemiplegia	36	(0.6)	64	(0.9)	26	(0.7)	46	(0.9)	10	(0.6)	18	(0.8)
Moderate or severe renal disease	299	(5.3)	430	(5.8)	203	(5.1)	298	(5.8)	96	(5.8)	132	(5.9)
Moderate or severe liver disease	6	(0.1)	11	(0.1)	3	(0.1)	8	(0.2)	3	(0.2)	3	(0.1)
No. of lymph node examined												
Unchecked	4,904	(87.3)	6,681	(90)	3,443	(87.2)	4,661	(90)	1,461	(87.6)	2,020	(89.9)
01–89	354	(6.3)	416	(5.6)	260	(6.6)	289	(5.6)	94	(5.6)	127	(5.7)
> = 90	311	(5.5)	296	(4.0)	216	(5.5)	206	(4.0)	95	(5.7)	90	(4.0)
Unknown	48	(0.9)	33	(0.4)	31	(0.8)	24	(0.5)	17	(1.0)	9	(0.4)
No. of lymph node invasive												
No invasive	120	(2.1)	178	(2.4)	86	(2.2)	124	(2.4)	34	(2)	54	(2.4)
1–89	255	(4.5)	252	(3.4)	189	(4.8)	176	(3.4)	66	(4)	76	(3.4)
> = 90	249	(4.4)	236	(3.2)	175	(4.4)	162	(3.1)	74	(4.4)	74	(3.3)
Unknown	4,993	(88.9)	6,760	(91)	3,500	(88.6)	4,718	(91.1)	1,493	(89.6)	2,042	(90.9)
Laterality												
Right	3,174	(56.5)	4,254	(57.3)	2,205	(55.8)	2,955	(57.0)	969	(58.1)	1,299	(57.8)
Left	2,340	(41.7)	3,114	(41.9)	1,674	(42.4)	2,186	(42.2)	666	(40.0)	928	(41.3)
Bilateral	50	(0.9)	36	(0.5)	39	(1.0)	25	(0.5)	11	(0.7)	11	(0.5)
Unspecified	53	(0.9)	22	(0.3)	32	(0.8)	14	(0.3)	21	(1.3)	8	(0.4)
Tumor stage												
IIIB	703	(12.5)	441	(5.9)	497	(12.6)	282	(5.4)	206	(12.4)	159	(7.1)
IV	4,914	(87.5)	6,985	(94.1)	3,453	(87.4)	4,898	(94.6)	1,461	(87.6)	2,087	(92.9)
Surgery (yes)	511	(9.1)	629	(8.5)	362	(9.2)	435	(8.4)	149	(8.9)	194	(8.6)
1st-line therapy												
TKI	-		5,979	(80.5)	-		4,168	(80.5)	0	(0)	1,811	(80.6)
Chemotherapy	5,469	(97.4)	1,359	(18.3)	3,856	(97.6)	953	(18.4)	1,613	(96.8)	406	(18.1)
Other treatment	148	(2.6)	88	(1.2)	94	(2.4)	59	(1.1)	54	(3.2)	29	(1.3)
Radiotherapy (yes)	1,735	(30.9)	2,135	(28.8)	1,181	(29.9)	1,507	(29.1)	554	(33.2)	628	(28.0)
ECOG performance status												
0	1,081	(19.2)	1,652	(22.2)	728	(18.4)	1,148	(22.2)	353	(21.2)	504	(22.4)
1	2,454	(43.7)	3,509	(47.3)	1,749	(44.3)	2,447	(47.2)	705	(42.3)	1,062	(47.3)
2	672	(12.0)	999	(13.5)	470	(11.9)	694	(13.4)	202	(12.1)	305	(13.6)
3	254	(4.5)	416	(5.6)	162	(4.1)	295	(5.7)	92	(5.5)	121	(5.4)
4	81	(1.4)	170	(2.3)	60	(1.5)	126	(2.4)	21	(1.3)	44	(2.0)
Unknown	1,075	(19.1)	680	(9.2)	781	(19.8)	470	(9.1)	294	(17.6)	210	(9.3)
Malignant Pleural Effusion												
No	2,364	(42.1)	3,661	(49.3)	1,641	(41.5)	2,586	(49.9)	723	(43.4)	1,075	(47.9)
Yes	2,025	(36.1)	3,278	(44.1)	1,431	(36.2)	2,280	(44.0)	594	(35.6)	998	(44.4)
Unknown	1,228	(21.9)	487	(6.6)	878	(22.2)	314	(6.1)	350	(21.0)	173	(7.7)
Smoking (yes)	3,090	(55.0)	2,062	(27.8)	2,157	(54.6)	1,428	(27.6)	933	(56.0)	634	(28.2)
Drinking (yes)	1,394	(24.8)	1,251	(16.8)	958	(24.3)	865	(16.7)	436	(26.2)	386	(17.2)
BMI												
BMI<18.5	381	(6.8)	521	(7.0)	269	(6.8)	368	(7.1)	112	(6.7)	153	(6.8)
18.5< = BMI<24	2,649	(47.2)	3,435	(46.3)	1,861	(47.1)	2,394	(46.2)	788	(47.3)	1,041	(46.3)
24< = BMI<27	1,191	(21.2)	1,716	(23.1)	820	(20.8)	1,216	(23.5)	371	(22.3)	500	(22.3)
27<BMI	693	(12.3)	1,061	(14.3)	482	(12.2)	735	(14.2)	211	(12.7)	326	(14.5)
Unknown	703	(12.5)	693	(9.3)	518	(13.1)	467	(9.0)	185	(11.1)	226	(10.1)

Abbreviation: EGFR (+): EGFR mutation positive; EGFR (-): EGFR wild-type; MI, Myocardial infarct; CHF, Congestive heart failure; PVD, Peripheral vascular disease; DM, Diabetes; BMI: body mass index.

### Independent prognostic factors in the training set

The most important predictors of the risk of death among patients with EGFR (+) were age (71–80 vs. < = 60: hazard ration [HR] = 1.18, 95% CI 1.08–1.29; >80 vs. < = 60: HR = 1.45, 95% CI 1.39–1.61), gender (male vs. female: HR = 1.18, 95% CI 1.10–1.28), CHF (yes vs. no: HR = 1.22, 95% CI 1.07–1.39), cerebrovascular disease (yes vs. no: HR = 1.12, 95% CI 1.01–1.23), moderate or severe renal disease (yes vs. no: HR = 1.18, 95% CI: 1.03–1.34), number of lymph node examined (unchecked vs. 1~89: HR = 1.28, 95% CI 1.03–1.58; > = 90 vs. 1~89: HR = 1.64, 95% CI 1.26–2.13), tumor stage (IV vs. IIIB: HR = 2.12, 95% CI 1.79–2.51), no surgery (no vs. yes: HR = 1.92, 95% CI 1.61–2.28), radiotherapy (yes vs. no: HR = 1.45, 95% CI 1.34–1.56), first-line therapy (chemotherapy vs. EGFR-TKI: HR = 1.18, 95% CI 1.09–1.28; other treatment vs. EGFR-TKI: HR = 1.50, 95% CI 1.14–1.97), ECOG performance status (ECOG PS 1 vs. 0: HR = 1.19, 95% CI 1.10–1.29; ECOG PS 2 vs. 0: HR = 1.68, 95% CI 1.51–1.87; ECOG PS 3 vs. 0: HR = 2.32, 95% CI 2.03–2.66; ECOG PS 4 vs. 0: HR = 3.09, 95% CI 2.55–3.74), had malignant pleural effusion (yes vs. no: HR = 1.41, 95% CI 1.32–1.50), and smoking (yes vs. no: HR = 1.11, 95% CI 1.02–1.21) (Table 2).

**Table 2:** Multivariable Cox proportional hazards regression analysis, point assignment and prognostic score: EGFR mutation positive patients.

	HR	(95% CI)	*P* Value	Point
Age				
< = 60 y	1.00			
61–70	1.00	(0.92–1.08)	0.9181	0
71–80	1.18	(1.08–1.29)	0.0001	15
>80 y	1.45	(1.30–1.61)	< .0001	33
Gender				
Female	1.00			
Male	1.18	(1.10–1.28)	< .0001	15
CHF				
No	1.00			
Yes	1.22	(1.07–1.39)	0.0025	18
Cerebrovascular disease				
No	1.00			
Yes	1.12	(1.01–1.23)	0.0249	10
Moderate or severe renal disease				
No	1.00			
Yes	1.18	(1.03–1.34)	0.0169	14
No. of lymph node examined				
01–89	1.00			
> = 90	1.64	(1.26–2.13)	0.0002	44
Unchecked	1.28	(1.03–1.58)	0.0232	22
Tumor stage				
IIIB	1.00			
IV	2.12	(1.79–2.51)	< .0001	67
Surgery				
Yes	1.00			
No	1.92	(1.61–2.28)	< .0001	58
Radiotherapy				
No	1.00			
Yes	1.45	(1.34–1.56)	< .0001	33
First-line therapy				
EGFR-TKI	1.00			
Chemotherapy	1.18	(1.09–1.28)	< .0001	15
Other treatment	1.50	(1.14–1.97)	0.0039	36
ECOG performance status				
0	1.00			
1	1.19	(1.10–1.29)	< .0001	15
2	1.68	(1.51–1.87)	< .0001	46
3	2.32	(2.03–2.66)	< .0001	75
4	3.09	(2.55–3.74)	< .0001	100
Malignant Pleural Effusion				
No	1.00			
Yes	1.41	(1.32–1.50)	< .0001	30
Smoking				
No	1.00			
Yes	1.11	(1.02–1.21)	0.0151	9

Abbreviation: HR, Hazard Ratio; CI, Confidence Interval; CHF, Congestive heart failure.

The most important predictors of the risk of death among EGFR (-) patients were age (61–70 vs. < = 60: HR = 1.08, 95% CI 0.99–1.18; 71–80 vs. < = 60: HR = 1.25, 95% CI 1.14–1.36; >80 vs. < = 60: HR = 1.45, 95% CI 1.28–1.64), gender (male vs. female: HR = 1.22, 95% CI 1.12–1.32), MI (yes vs. no: HR = 2.06, 95% CI 1.53–2.78), cerebrovascular disease (yes vs. no: HR = 1.12, 95% CI 1.00–1.25), chronic pulmonary disease (yes vs. no: HR = 1.12, 95% CI 1.04–1.20), number of lymph node examined (unchecked vs. 1~89: HR = 1.18, 95% CI 0.98–1.42; > = 90 vs. 1~89: HR = 1.47, 95% CI 1.16–1.86), tumor stage (IV vs. IIIB: HR = 1.38, 95% CI 1.23–1.55), no surgery (no vs. yes: HR = 2.08, 95% CI 1.76–2.45), radiotherapy (yes vs. no: HR = 1.24, 95% CI 1.15–1.35), ECOG performance status (ECOG PS 1 vs. 0: HR = 1.26, 95% CI 1.15–1.37; ECOG PS 2 vs. 0: HR = 1.71, 95% CI 1.52–1.92; ECOG PS 3 vs. 0: HR = 2.01, 95% CI 1.71–2.37; ECOG PS 4 vs. 0: HR = 3.01, 95% CI 2.34–3.88), presentation of malignant pleural effusion (yes vs. no: HR = 1.25, 95% CI 1.16–1.35), and smoking history (yes vs. no: HR = 1.26, 95% CI 1.16–1.37) (Table 3).

**Table 3:** Multivariable Cox proportional hazards regression analysis, point assignment and prognostic score: EGFR wild-type patients.

	HR	(95% CI)	*P* Value	Point
Age				
< = 60 y	1.00			0
61–70	1.08	(0.99–1.18)	0.0682	7
71–80	1.25	(1.14–1.36)	< .0001	20
>80 y	1.45	(1.28–1.64)	< .0001	34
Gender				
Female	1.00			0
Male	1.22	(1.12–1.32)	< .0001	18
MI				
No	1.00			0
Yes	2.06	(1.53–2.78)	< .0001	65
Cerebrovascular disease				
No	1.00			
Yes	1.12	(1.00–1.25)	0.0489	10
Chronic pulmonary disease				
No	1.00			0
Yes	1.12	(1.04–1.20)	0.0024	10
No. of lymph node examined				
01–89	1.00			0
> = 90	1.47	(1.16–1.86)	0.0012	35
Unchecked	1.18	(0.98–1.42)	0.0811	15
Tumor stage				
IIIB	1.00			0
IV	1.38	(1.23–1.55)	< .0001	29
Surgery				
Yes	1.00			0
No	2.08	(1.76–2.45)	< .0001	66
Radiotherapy				
No	1.00			0
Yes	1.24	(1.15–1.35)	< .0001	20
ECOG performance status				
0	1.00			0
1	1.26	(1.15–1.37)	< .0001	21
2	1.71	(1.52–1.92)	< .0001	49
3	2.01	(1.71–2.37)	< .0001	63
4	3.01	(2.34–3.88)	< .0001	100
Malignant Pleural Effusion				
No	1.00			0
Yes	1.25	(1.16–1.35)	< .0001	21
Smoking				
No	1.00			0
Yes	1.26	(1.16–1.37)	< .0001	21

Abbreviation: HR, Hazard Ratio; CI, Confidence Interval; MI, myocardial infarction.

We compared the R^2^ and AIC of different models with the null model by adding significant prognostic factors one at a time (**S1 Table**). The final nomogram model demonstrated the best fit and identified 13 variables for EGFR (+) and 12 variables for EGFR (-) that had strongest association with OS risk. BETAs of each variable were estimated and converted to a score. In the EGFR (+) group, the ECOG performance status had the highest BETA and was assigned 100 points on the scale (when ECOG performance score = 4), and the remaining variables were assigned a smaller number of points proportional to their effect size.

### Nomogram for 1-year and 2-year overall survival

**Fig 1:**
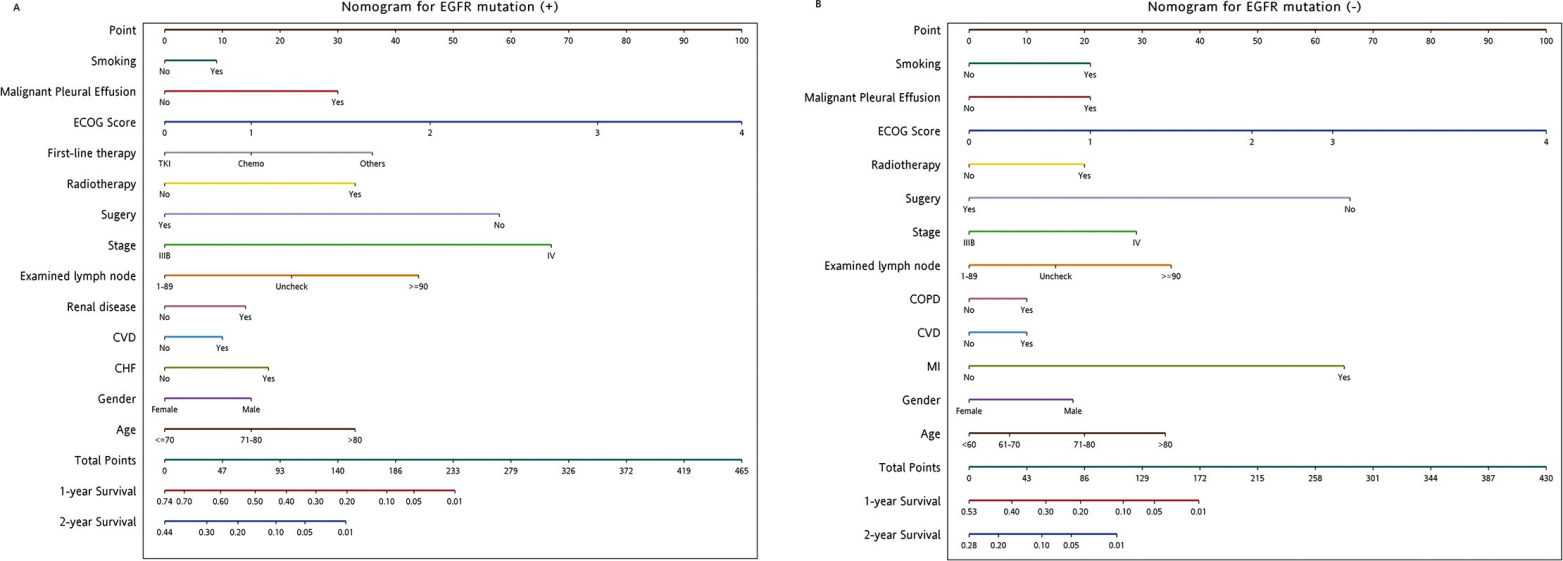
Prediction probability nomograms (1-year and 2-year). (A) EGFR mutation positive (EGFR(+)) patients. (B) EGFR wide-type (EGFR(-)) patients.

The nomograms were developed for prediction of OS and can assign numeric predictions points for the risk of death at 1 and 2-years (Fig 1A and Fig 1B). Higher total points based on the sum of the assigned number of points for each factor in the nomograms were associated with a worse prognosis. In using the proposed nomogram, each subtype within these variables was assigned a score, which was then totaled and located on a point scale. This made it possible to estimate the probability of 1-year and 2-year risk of death simply by drawing a straight line. For example, in the EGFR(+) nomogram, if a male patient (point: 15), with CHF (point: 18), received surgery (point: 0), received radiotherapy (point: 33), ECOG performance status was 2 (point: 46), had no malignant pleural effusion (point: 0), with EGFR-TKI as first-line treatment (point: 0) and no smoking history (point: 0), for a predicted 1-year and 2-year OS of 34% and <10%, respectively. Similarly, a 60 years old female patient who had tumor stage III in EGFR(-), had cerebrovascular disease history, received surgery, received no radiotherapy, ECOG performance status was 1 and without malignant pleural effusion would have a total of 31 points (0 point for patient’s age, 0 points for male, 10 points for cerebrovascular disease, 0 point for tumor stage III, 0 point for surgery, 21 for ECOG performance status, 0 point for malignant pleural effusion). For this patient, the predicted 1-year OS was 40% and the predicted 2-year OS was 10%.

### Calibration and validation of nomograms

**Fig 2:**
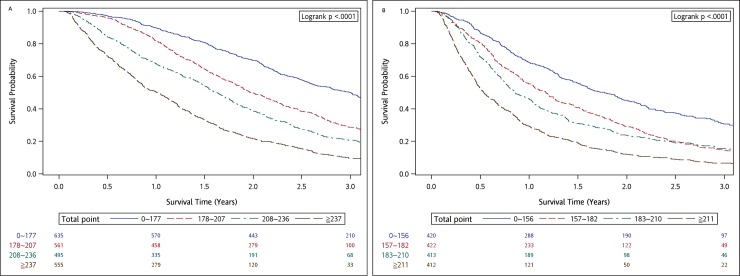
Survival probability (according to quartiles of total points) for 1-year survival. (A) EGFR mutation positive (EGFR(+)) patients. (B) EGFR wild-type (EGFR(-)) patient. The quartiles of EGFR mutation positive patients is defined as follows: quartile 1: 0 to 177 points; quartile 2: 178–207 points; quartile 3: 208–236 points; quartile 4: ≧237 points. The quartiles of EGFR wild-type patients defined as follows: quartile 1: 0 to 156 points; quartile 2: 157–182 points; quartile 3: 183–210 points; quartile 4:≧211 points.

To further assess the discriminative ability of the models, the predicted probability of 1-year and 2-year OS was then plotted as Kaplan-Meier curves stratified by quartiles of total points of the predicted probability calculated from the nomograms (Fig 2A and 2B). Patients with the lowest predicted 1-yer OS (quartiles 4) had a substantially worse outcome (29.37% 1-year survival for EGFR(-) and 50.27% 1-year survival for EGFR(+)) compared with patients in quartiles 1, 2 and 3 (S2 Table).

**Fig 3:**
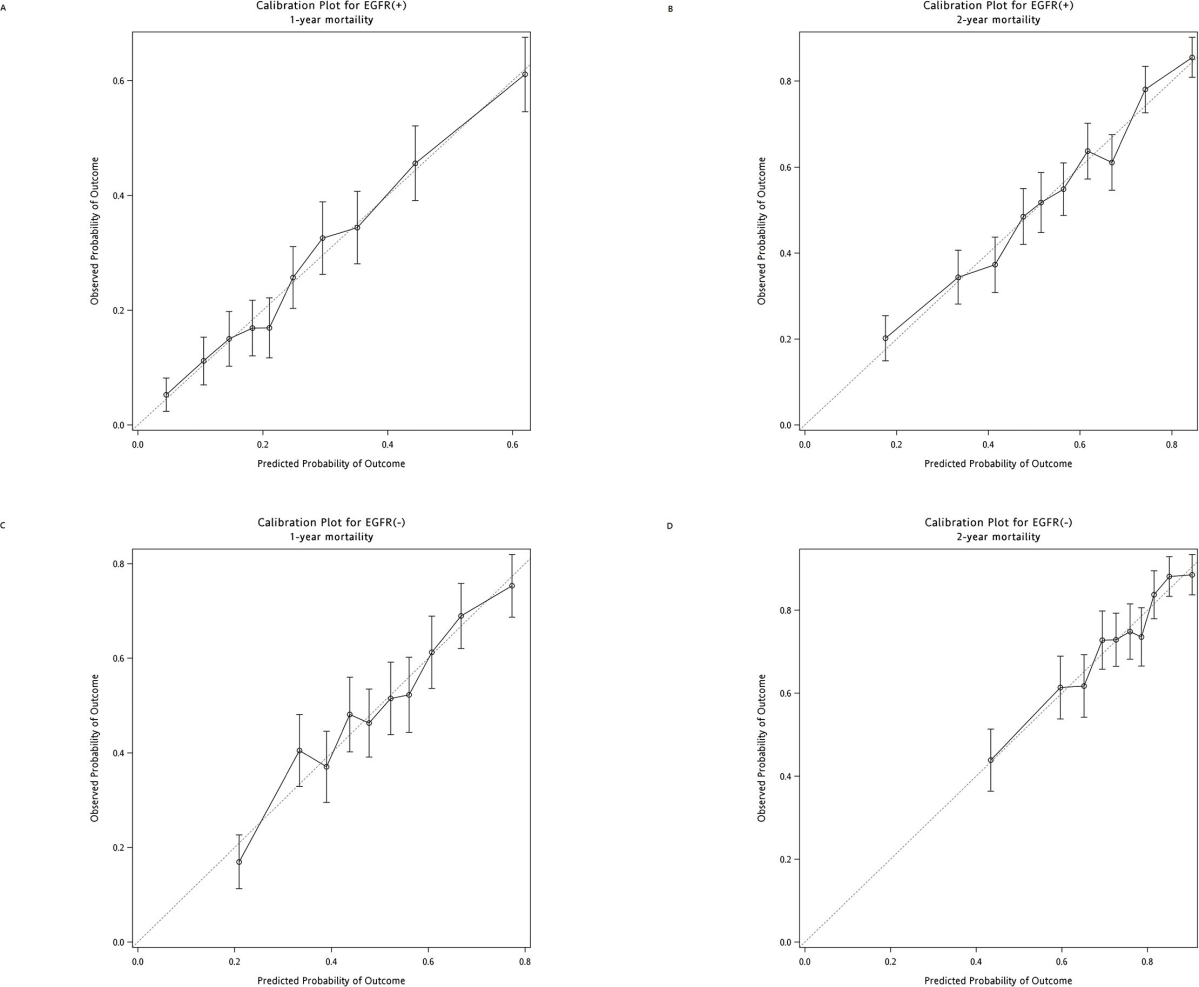
Calibration of nomograms. **Calibration curves of the nomogram.** (A) 1-year survival probability of EGFR(+) patients. (B) 2-year survival probability of EGFR (+) patients. (C) 1-year probability of EGFR(-) patients. (D) 2-year survival probability of EGFR (-) patient. The calibration curves were close to the 45-degres line.

The observed and predicted probability of OS was then plotted as calibration plots stratified by 10 percentile of the predicted probability calculated from the nomograms. Both the calibration plots for EGFR(+) patients (Fig 3A and 3B) and for EGFR(-) patients (Fig 3C and 3D) were well matched the ideal 45-degree line and showed good correlation between predicted and observed outcomes. The discriminative ability of the final model for 1-year and 2-year was also assessed using the C-statistics. The C-statistics for the prediction of OS for EGFR(+) patients was 0.83 (95% CI: 0.80–0.87), and 0.88 (95% CI: 0.85–0.91) for EGFR(-) patients.

## Discussion

Tumor EGFR mutation status is an important variable in the selection of regimens for patients with advanced NSCLC. EGFR mutation leads to constitutive activation of the receptor, independent of ligand binding. They are also associated with increased sensitivity to the specific EGFR TKIs such as gefitinib, erlotinib, and afatinib. The response rate of these agents is highly associated with progression-free survival and overall survival. Previous studies have adopted nomograms to establish prognostic models for NSLCL patients; however, those previous models did not include information related to EGFR mutation status.[8, 9, 25–28] Accurate prognostication for advanced NSCLC is important not only to select patients for treatments but also to inform patients accurately about their long-term prognosis.

To the best of our knowledge, this is the first nomogram combining an important molecular factor to predict survival probability among patients with advanced adenocarcinoma, based on a large database with long-term follow-up. Linking the point score and total score of each predictor makes it possible to estimate the patient survival within a specific span of time. Identifying subgroups of patients according to their risk of death could be very helpful to physicians and patients alike in the selection of treatment regiments to improve survival. At present, the administration of additional treatments and whether to implement intensive follow-up remain issues of controversy.[29]

Our study cohort was obtained from a national cancer registry database. The large sample size of the cohort ensured that it is generalizable for Asian populations with advanced adenocarcinoma. Multivariable analysis identified gender, surgical treatment, radiotherapy, ECOG performance status and malignant pleural effusion are independent prognostic factors among patients with EGFR mutation positive as well as those with EGFR wild-type. These findings are in agreement with previous reports on risk factors for NSCLC.[9, 14, 27, 30–33] Nonsmoking history has been identified as a prognostic indicator in many studies.[34–36] Our results revealed that for patients with either EGFR mutation positive or wild-type, the association between smoking status and survival remains significant in the presence of strong predictors, such as treatment and ECOG performance scores. Unlike previous studies,[26, 37, 38] lymph node invasion was not a significant predictor in this study. This may be due to the fact that more than 88% of the patients in our sample presented involvement of ≧ 90 lymph nodes. Putila and Guo reported that among patients with adenocarcinoma, COPD, congestive heart failure, peripheral vascular disease, cerebrovascular disease, diabetes with complications, and gastrointestinal ulcers are associated with a significantly higher risk of death.[39] Similar findings were obtained in the current study. For either EGFR mutation positive or wild-type patients with cerebrovascular disease is associated with a higher risk of death.

Accurate prognostic tools can help physicians and patients reach the consensus during the decision-making process with regard to the treatment and management of disease. Nomogram validation is required to avoid model overfitting and determine its generalizability.[40] The advantages of the proposed model is that the value of variables in the model can be obtained through routine clinical practice. Furthermore, our model is capable of achieving prediction accuracy and discriminant accuracy far exceeding those of the TNM staging system[41, 42], as indicated by the C-index. Unlike existing tools using participants in clinical trials, our model was derived from known outcomes in the context of a National Health Insurance system. To satisfy the inclusion criteria of most RCTs, patients must be highly homogeneous and present defined characteristics (e.g., specific tumor stage and age groups). The homogeneity of patient groups in many of these clinical trials renders the models largely inapplicable in a real-world setting. Even in cases where the models meet external validation, the number of patients in the development and validation cohorts were relatively small.[6, 7, 43, 44] In contrast, our model derived from a nationwide cancer registry and healthcare dataset is applicable to a wide range of patient populations in the real-world practice.

Nonetheless, our study could be further improved. Progress in the testing of molecular markers has made it far easier to integrate molecular profiles in clinical use. Currently, our model dealt only with EGFR mutation status. We expect that the inclusion of other well-established markers such as anaplastic lymphoma kinase (ALK)-EML4 fusion, would further enhance prediction accuracy. Second, the proposed nomogram is limited by the retrospective nature of the data collection and did not incorporate a number of recognized prognostic parameters (e.g., tumor location and tumor size).[45] The model could be further refined by improving prospective data collection and patient follow-up and/or including other factors. Future study could look at the impact of using these nomograms on early selection of treatments and subsequently influence outcomes. Third, we excluded those patients with missing values and did not consider the interaction between predictors which may underestimate the effect of each predictor. Fourth, our findings were estimated based on the Taiwan-based database which may limit the generalizability to other countries. Finally, this study focused only on advanced stage adenocarcinoma patients. Further research incorporating a wider range of patients would no doubt be more beneficial.

To conclude, we established and validated two separate nomograms for the different EGFR mutation status. This model makes it possible to estimate the risk of death of patients more precisely and can identify subgroups of patients who are in need of specific treatment strategies and consequently, may improve their survival.

## Supporting information

S1 Fig Survival probability for 1-year survival between EGFR mutation status. (A) among patients of training group and (B) among patients of validation group.
(PDF)

S1 Table Goodness-of-fit of prognostic factors. (PDF)

S2 Table The 1-year and 2-year survival among study cohorts. (PDF)
