# Supplementary material for: Development and validation of nomograms for predicting survival probability of patients with advanced adenocarcinoma in different EGFR mutation status
Source: PLoS One. 2019 Aug 16;14(8):e0220730. doi: 10.1371/journal.pone.0220730 (PMC6697331; doi:10.1371/journal.pone.0220730)
Supplement: S1 Fig — Survival probability for 1-year survival between EGFR mutation status. (A) among patients of training group and (B) among patients of validation group. (PDF) [file pone.0220730.s001.pdf]

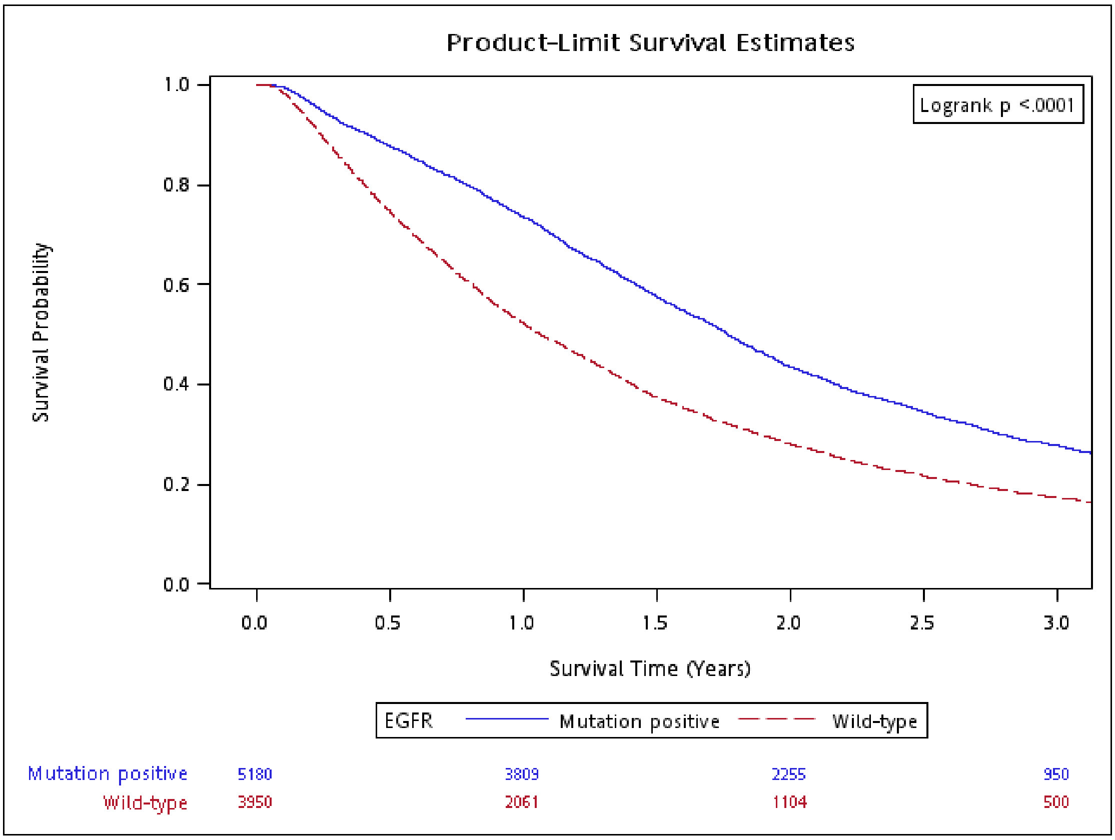

S1 Fig A

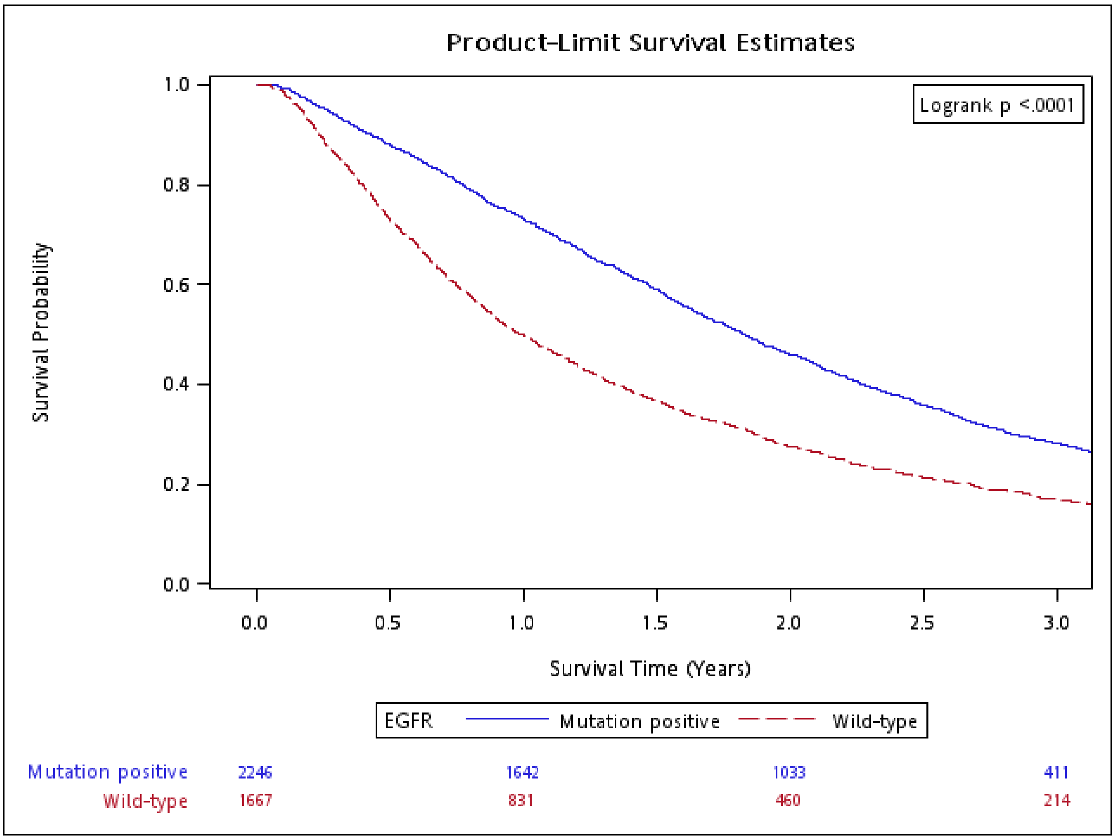

S1 Fig B

S1 Fig. Survival probability for 1-year survival between EGFR mutation status. (A) among patients of training group and (B) among patients of validation group
