# Supplementary material for: Development and validation of nomograms for predicting survival probability of patients with advanced adenocarcinoma in different EGFR mutation status
Source: PLoS One. 2019 Aug 16;14(8):e0220730. doi: 10.1371/journal.pone.0220730 (PMC6697331; doi:10.1371/journal.pone.0220730)
Supplement: S1 Table — (PDF) [file pone.0220730.s002.pdf]

S2 Table. Goodness-of-fit of prognostic factors

		AIC	Likelihood -ratio (LR)	Individual variable likelihood-ratio	Generalized R^2	Residual Analysis	
						Chi-square	*P value*
**EGFR(+) (n=5,180)**							
Null model		62,394.99				1,118.42	<.0001
Model 1	ECOG performance status	61,920.51	482.48	482.48	0.089	548.35	<.0001
Model 2	Surgery	61,720.22	684.76	202.29	0.124	387.01	<.0001
Model 3	Malignant Pleural Effusion	61,654.15	752.84	68.08	0.135	320.02	<.0001
Model 4	Radiotherapy	61,592.28	816.70	63.86	0.146	256.70	<.0001
Model 5	Tumor stage	61,520.69	890.29	73.59	0.158	197.23	<.0001
Model 6	Age	61,457.65	959.34	69.04	0.169	124.90	<.0001
Model 7	Gender	61,407.76	1,011.23	51.89	0.177	71.86	<.0001
Model 8	First-line therapy	61,390.79	1,032.19	20.97	0.181	49.62	0.0004
Model 9	No. of lymph node examined	61,381.07	1,045.92	13.73	0.183	35.43	0.0124
Model 10	CHF	61,373.60	1,055.39	9.47	0.184	25.34	0.1158
Model 11	Moderate or severe renal disease	61,370.10	1,060.88	5.50	0.185	19.55	0.2978
Model 12	Smoking	61,366.41	1,066.57	5.69	0.186	13.84	0.6109
Model 13	Cerebrovascular disease	61,363.50	1,071.48	4.91	0.187	8.81	0.8874
**EGFR(-) (n=3,950)**							
Null model		51,164.61				776.66	<.0001
Model 1	ECOG performance status	50,908.72	263.89	263.89	0.065	487.77	<.0001
Model 2	Surgery	50,690.29	484.32	220.43	0.115	309.76	<.0001
Model 3	Smoking	50,613.03	563.57	79.25	0.133	229.29	<.0001
Model 4	Age	50,567.89	614.72	51.15	0.144	175.03	<.0001
Model 5	Tumor stage	50,523.15	661.45	46.73	0.154	131.61	<.0001
Model 6	MI	50,506.68	679.93	18.47	0.158	107.76	<.0001
Model 7	Gender	50,486.92	701.69	21.76	0.163	86.34	<.0001
Model 8	Malignant Pleural Effusion	50,466.50	724.11	22.42	0.167	64.36	<.0001
Model 9	Radiotherapy	50,439.09	753.52	29.41	0.174	34.10	0.0179
Model 10	No. of lymph node examined	50,432.40	764.21	10.69	0.176	23.05	0.1475
Model 11	Chronic pulmonary disease	50,425.77	772.83	8.62	0.178	14.42	0.5673
Model 12	Cerebrovascular disease	50,424.00	776.61	3.78	0.178	10.52	0.7861
