# Supplementary material for: Development and validation of nomograms for predicting survival probability of patients with advanced adenocarcinoma in different EGFR mutation status
Source: PLoS One. 2019 Aug 16;14(8):e0220730. doi: 10.1371/journal.pone.0220730 (PMC6697331; doi:10.1371/journal.pone.0220730)
Supplement: S2 Table — (PDF) [file pone.0220730.s003.pdf]

S3 Table. The 1-year and 2-year survival among study cohort

Total point (quartile)	Training Set			Validation Set		
	N	No. of Death	Survival rate	N	No. of Death	Survival rate
**1-year Survival**						
**EGFR(+)**						
0~177	1,346	152	88.71%	635	65	89.76%
178~207	1,320	259	80.38%	561	103	81.64%
208~236	1,218	344	71.76%	495	160	67.68%
≧237	1,296	615	52.55%	555	276	50.27%
**EGFR(-)**						
0~156	1,132	304	73.14%	420	132	68.57%
157~182	996	423	57.53%	422	189	55.21%
183~210	972	552	43.21%	413	222	46.25%
≧211	850	604	28.94%	412	291	29.37%
**2-year Survival**						
**EGFR(+)**						
0~177	1,346	445	66.94%	635	192	69.76%
178~207	1,320	691	47.65%	561	282	49.73%
208~236	1,218	762	37.44%	495	304	38.59%
≧237	1,296	1,024	20.99%	555	435	21.62%
**EGFR(-)**						
0~156	1,132	594	47.53%	420	230	45.24%
157~182	996	694	30.32%	422	300	28.91%
183~210	972	784	19.34%	413	315	23.73%
≧211	850	769	9.53%	412	362	12.14%
